# Analyzing nonlinear oscillations with He's frequency-amplitude method and numerical comparison in jet engine vibration system

**DOI:** 10.1016/j.heliyon.2024.e24261

**Published:** 2024-01-07

**Authors:** M. Abul Kawser, Md. Abdul Alim, Nazmul Sharif

**Affiliations:** a Department of Mathematics, Islamic University, Kushtia, Bangladesh; b Department of Mathematics, Rajshahi University of Engineering & Technology, Rajshahi, Bangladesh

**Keywords:** He's frequency-amplitude method (HFAM), Jet engine vibration system, Nonlinear oscillators, Damp and force system

## Abstract

In this study, we describe and successfully solve a jet engine vibration equation using a straightforward tool known as the He's frequency-amplitude Method (HFAM). The jet engine vibration system demonstrates diverse applications across aerospace, power generation, industrial machinery, transportation, marine propulsion, energy optimization, defense, and aviation training. Utilizing HFAM, we derive periodic solutions in a general form for this system, considering various cases dependent on damping and driving forces. The obtained results highlight the effectiveness of HFAM as a distinct and straightforward technique for nonlinear equations. By comparing the solutions with numerical results obtained using the fourth-order Runge-Kutta method, we demonstrate the excellent accuracy of our solutions.

## 1 Introduction

In engineering and mathematics, nonlinear oscillation has emerged as an increasingly significant and captivating area of interest. Solving nonlinear differential equations is generally more challenging than solving linear ones, particularly when it comes to nonlinear damping and force systems. Consequently, various cutting-edge methods are being harnessed to approximate solutions for nonlinear conundrums. Notably, certain methodologies, including the variational iteration method [1,2], energy balance method [3], homotopy perturbation method (HPM) [4,5], Adomian decomposition method [6], the Lindstedt–Poincaré method [7], the finite element method [8], the boundary element method [9], the spectral method [10], the finite difference method [11], the He's frequency-amplitude method (HFAM) [[12], [13], [14], [15], [16], [17]] and more others have established their significance.

The Chinese mathematician Ji-Huan He made the first significant development of HFAM. A significant advancement has made in He's formulation for nonlinear oscillators [18]. Two-scale fractal calculus was introduced to the Zhiber-Shabat oscillator [19] with the help of the frequency formulation, while the frequency formulation was optimized for the tangent oscillator [20] and subsequently extended to the fractal Duffing oscillator [21]. Moreover, an innovative modification has introduced to the frequency-amplitude formulation, which is specifically designed for fractal vibration systems [22]. Meanwhile, the Hamiltonian-based approach [23] was optimized, and, in a distinct research initiative, the exploration of forced nonlinear oscillators in a fractal space [24] was conducted, employing He's frequency formulation. These initiatives have a profound impact on furthering our comprehension and the practical implementation of He's inventive frequency-amplitude formulation in the dynamic landscape of nonlinear oscillators.

In this study, we utilized the HFAM to address the problem of jet engine vibration. Recently, Kawser et al. [25] employed the HPM to successfully address the problem. They derived solutions for specific numeric values of the parameters of the equation. In this research, we extend their work by deriving the solutions in general forms. That is, in this article all the parameters exist in the solutions in symbolic form rather than numerically. Although HPM usually gives correct solutions, it has limitations and sometimes fails to provide answers. In particular, for any particular value of the parameters the results may deviate significantly from the solution direction. In contrast, HFAM offers a straightforward calculation process that consistently provides elegant and satisfactory solutions in general form. The HPM have been applied to address the forced term in electrically actuated microbeam-based MEMS [26] and the axial vibration of strings [27], however our study has focused on analyzing the effect of the forced term on the jet engine model using the HFAM.

The equations of jet engine vibration play a critical role in ensuring the structural integrity, safety, and performance of aircraft engines. These equations are used to analyze and predict vibrations caused by various factors, such as engine imbalance, aerodynamic forces, and mechanical interactions. By understanding and mitigating engine vibrations, engineers can enhance the reliability and longevity of jet engines, reduce maintenance costs, and ultimately improve the safety and comfort of air travel.

**Fig. 1:**
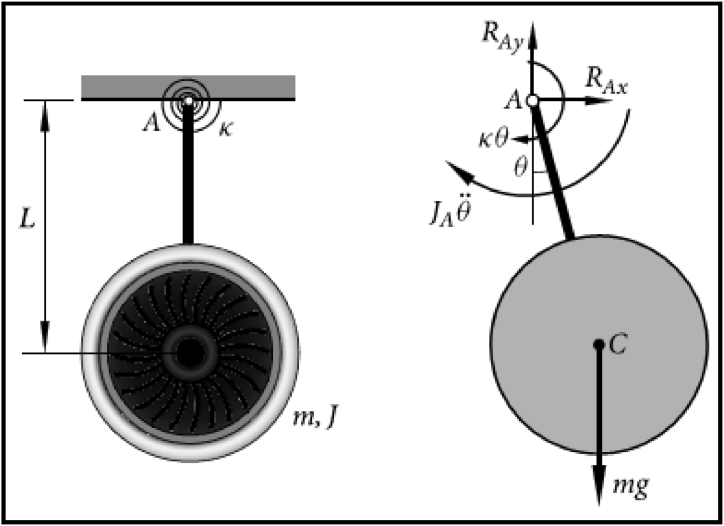
Jet engine vibration model.

Here a moment of inertia denoted as J is associated with the axis of the center of gravity C and the mass of the engine is represented by m. In the simulation of the horizontal movement of a jet engine, an elastic beam is utilized to provide support for a rigid body. In this scenario, a weightless rod is employed, pivotally attached at point A. The rotational spring constant is denoted as κ, which results in the application of a restoring moment defined as κθ. The length of the rod is represented as L, and in Fig. 1, θ signifies the angle formed between the rod and the vertical reference line. In the case of small rotations, that is |θ|≪1, one can establish the equation of motion for the jet engine by incorporating the θ parameter to ascertain the frequency of its natural vibration.

The hinge in the system revolves at point A and the jet engine has a moment of inertia, denoted as J, around its center of mass at point C. Applying the parallel axis theorem, the moment of inertia around axis A for the jet engine is:

(1) $$J_{A}=J+mL^{2}\text{.}$$

Gravity mg acts on the jet engine. We represent the reaction force at A as two components, $R_{Ax}$ and $R_{Ay}\text{.}$ As the system exhibits counterclockwise angular changes in both angle θ and angular acceleration $\ddot{\theta }\text{,}$ so the rotating spring exerts clockwise restoring moment κθ and inertia moment $J_{A}\ddot{\theta }$ respectively. Since the free body in Fig. 1 is in a state of dynamic equilibrium, so the D'Alembert's Principle gives.

$$J_{A}\ddot{\theta }+\kappa \theta +mgL\;\sin\;\theta =0\text{.}$$

(2) $$\text{That}\;\text{is}\;\frac{J+mL^{2}}{L}\ddot{\theta }+\frac{\kappa }{L}\theta +mg\;\sin\;\theta =0\text{.}$$

Since damping force is directly proportional to velocity and acts in the opposite direction. So, equation (2) is modified by adding the viscous damping term $-2\delta \dot{\theta }\text{,}$ with δ denoting the damping coefficient. Thus, the equation of jet engine vibration takes the form:

$$\frac{J+mL^{2}}{L}\ddot{\theta }+\frac{\kappa }{L}\theta +mg\;\sin\;\theta =-2\delta \dot{\theta }\text{.}$$

(3) $$\text{That}\;\text{is}\;\frac{J+mL^{2}}{L}\ddot{\theta }+2\delta \dot{\theta }+\frac{\kappa }{L}\theta +mg\;\sin\;\theta =0\text{.}$$

Now introducing the model with the supplementary periodic external force Fcosλt, then the model becomes:

(4) $$\frac{J+mL^{2}}{L}\ddot{\theta }+2\delta \dot{\theta }+\frac{\kappa }{L}\theta +mg\;\sin\;\theta -F\;\cos\;\lambda t=0\text{.}$$

Expanding sinθ in a power series of θ and then neglecting terms higher than $\theta^{3}\text{,}$ due to θ≪1, the nonlinear jet engine vibration equation is derived as follows:

(5) $$\frac{J+mL^{2}}{L}\ddot{\theta }+2\delta \dot{\theta }+\left(\frac{\kappa }{L}+mg\right)\theta -\frac{1}{6}mg\theta^{3}-F\;\cos\;\lambda t=0\text{.}$$

## 2 Methodology

Consider the following nonlinear differential oscillator:

(6) $$x^{\prime \prime }+f\left(x\right)=0,x\left(0\right)=A,x^{\prime }\left(0\right)=0\text{.}$$

According to HFAM [[28], [29], [30]], the frequency of equation (6) can be expressed as follows:

(7) $$\omega^{2}={\frac{df\left(x\right)}{dx}|}_{x=A/2}\text{.}$$

And the approximate analytical periodic solution is given by

(8) x(t)=Acosωt.

## 3 Solution procedure

### 3.1 In the absence of damping and driving forces

Firstly, we consider the following nonlinear oscillator in the absence of damping and driving forces, as represented by equation (5):

(9) $$\left(\frac{J+mL^{2}}{L}\right)\ddot{\theta }+\left(\frac{\kappa }{L}+mg\right)\theta -\frac{1}{6}mg\theta^{3}=0,\theta \left(0\right)=A,\dot{\theta }\left(0\right)=0\text{.}$$

Here $f\left(\theta \right)=\frac{L}{J+mL^{2}}\left\{\left(\frac{\kappa }{L}+mg\right)\theta -\frac{1}{6}mg\theta^{3}\right\}$.

Hence based on HFAM [[16], [17], [18]], we have

(10) $$\omega^{2}=\frac{L}{J+mL^{2}}\left\{\left(\frac{\kappa }{L}+mg\right)-\frac{1}{8}mgA^{2}\right\}\text{.}$$

Therefore, as like solution (8) we obtained the following periodic solution for equation (9):

(11) θ(t)=Acosωt.

### 3.2 In the presence of damping and absence of driving forces

Suppose the damped nonlinear oscillator without any driving forces, based on equation (5):

(12) $$\left(\frac{J+mL^{2}}{L}\right)\ddot{\theta }+2\delta \dot{\theta }+\left(\frac{\kappa }{L}+mg\right)\theta -\frac{1}{6}mg\theta^{3}=0,\theta \left(0\right)=A,\dot{\theta }\left(0\right)=0\text{.}$$

According to HFAM, the natural frequency $\omega^{2}$ is determined as equation (10).

Now, equation (12) becomes to a linear damping jet engine vibration equation. Thus, the solution takes the form:

(13) $$\theta \left(t\right)=Ae^{-\left(\frac{\delta L}{J+mL^{2}}\right)t}\;\cos\;\Omega t\text{.}$$

In this context, $\Omega =\sqrt{\omega^{2}-\delta^{2}{\left(\frac{L}{J+mL^{2}}\right)}^{2}}$ is a non-conservative frequency.

### 3.3 In the absence of damping and presence of driving forces

Assumed a nonlinear oscillator with driving forces in the absence of damping of the following form:

(14) $$\left(\frac{J+mL^{2}}{L}\right)\ddot{\theta }+\left(\frac{\kappa }{L}+mg\right)\theta -\frac{1}{6}mg\theta^{3}-F\;\cos\;\lambda t=0,\theta \left(0\right)=A,\dot{\theta }\left(0\right)=0\text{.}$$

In this situation $\omega^{2}$ is defined by equation (10).

The solution of equation (14) for this phenomenon is obtained as follows:

(15) θ(t)=(A−α)cosωt+αcosλt.

Herein, $\alpha =\frac{FL}{J+mL^{2}}\left(\frac{1}{\omega^{2}-\lambda^{2}}\right)$.

### 3.4 In the presence of damping and driving forces

Finally, consider a nonlinear oscillator with damping and driving forces of the following form:

(16) $$\left(\frac{J+mL^{2}}{L}\right)\ddot{\theta }+2\delta \dot{\theta }+\left(\frac{\kappa }{L}+mg\right)\theta -\frac{1}{6}mg\theta^{3}-F\;\cos\;\lambda t=0,\theta \left(0\right)=A,\dot{\theta }\left(0\right)=0\text{.}$$

Under this condition $\omega^{2}$ is given by equation (10).

In this case, the solution of equation (16) takes the form:

(17) $$\theta \left(t\right)=\left\{A-F\gamma \left(\frac{\sigma }{4\lambda^{2}\delta^{2}\gamma^{2}+\sigma^{2}}\right)\right\}e^{-\delta \gamma t}\;\cos\;\Omega t+F\gamma \left(\frac{2\delta \lambda \gamma \;\sin\;\lambda t+\sigma \;\cos\;\lambda t}{4\lambda^{2}\delta^{2}\gamma^{2}+\sigma^{2}}\right)\text{.}$$

Herein, $\sigma =\omega^{2}-\lambda^{2},\gamma =\frac{L}{J+mL^{2}}$ and $\Omega =\sqrt{\omega^{2}-\delta^{2}{\left(\frac{L}{J+mL^{2}}\right)}^{2}}$ is a non-conservative frequency.

## 4 Results and discussion

**Fig. 2:**
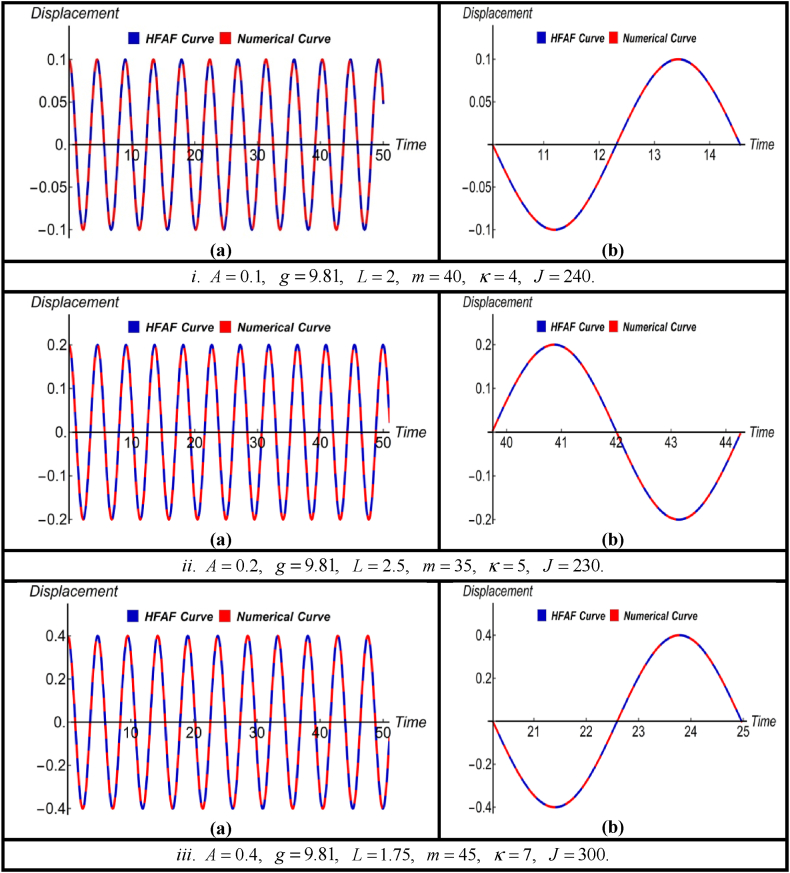
(a) Comparison between HFAM outcomes with numerical findings for Case-I and (b) close-up view of a specific section of (a).

**Fig. 3:**
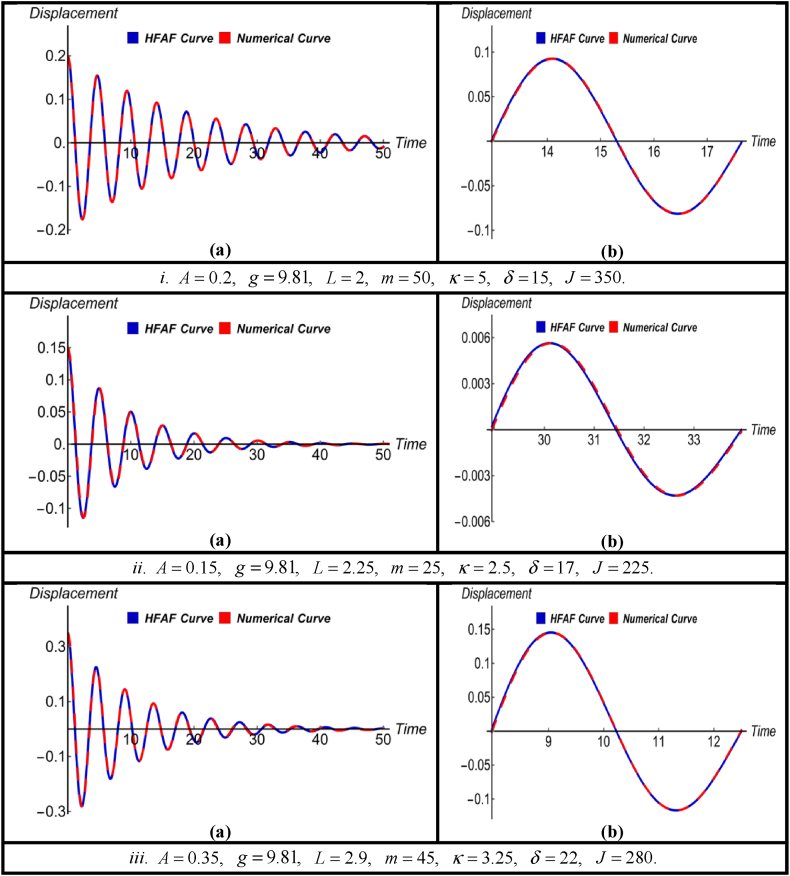
(a) Comparison between HFAM outcomes with numerical findings for Case-II and (b) close-up view of a specific section of (a).

**Fig. 4:**
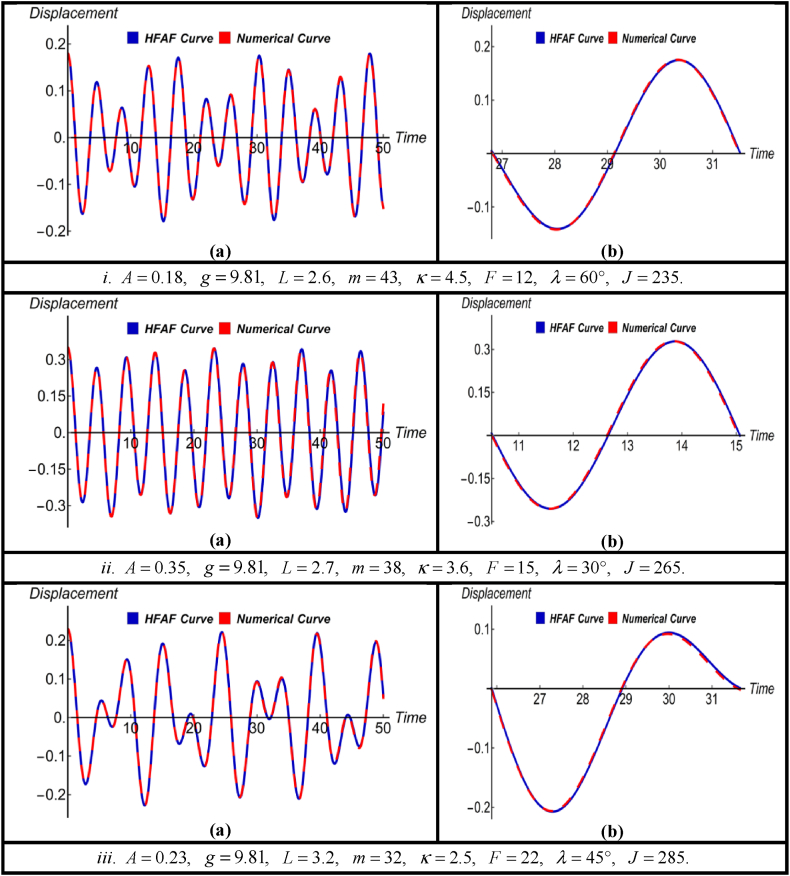
(a) Comparison between HFAM outcomes with numerical findings for Case-III and (b) close-up view of a specific section of (a).

**Fig. 5:**
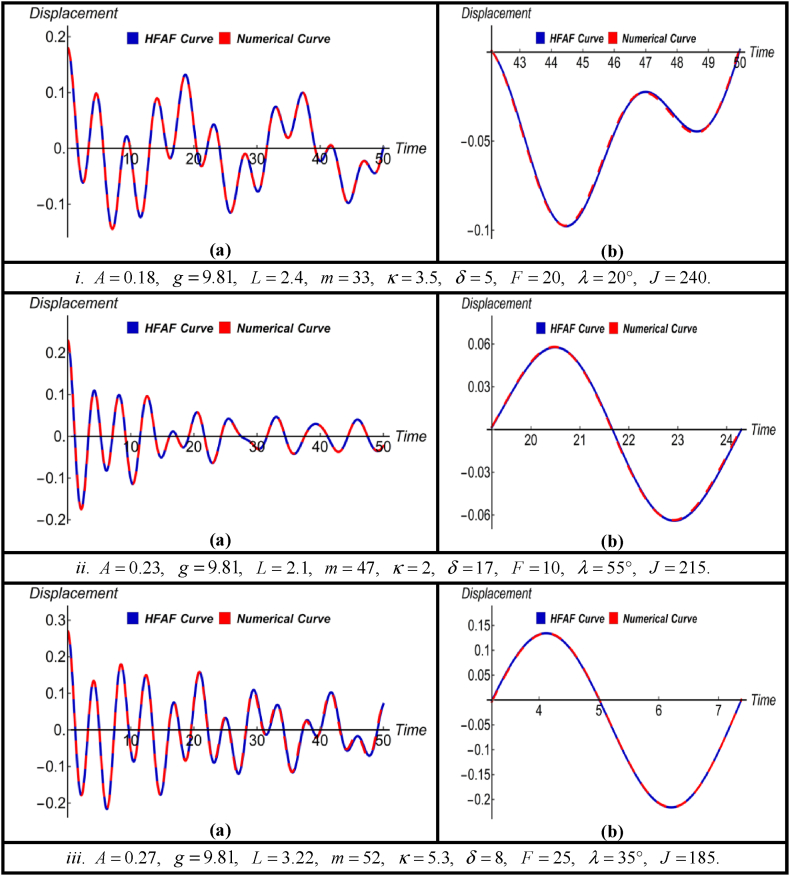
(a) Comparison between HFAM outcomes with numerical findings for Case-IV and (b) close-up view of a specific section of (a).

The nonlinear jet engine vibration system has been effectively addressed using the HFAM, providing a solution in its general form without the need to consider specific parameter values. This approach encompasses various parameters, including nonlinear constants, rotating spring constants, damping constants, driving forces, and more. Notably, parametric initial conditions have also been integrated into this approach during the system-solving process. Fig. 2, Fig. 3, Fig. 4, Fig. 5 illustrate graphical comparisons between the numerical results and the solutions obtained using the aforementioned method, thereby demonstrating its effectiveness and validity. For each solution, three graphical representations are provided for three different sets of parameter values within the equation and all the values of the parameters are taken in the international system (SI) of units. To ensure the accuracy of the findings, an enlarged view of a specific portion within each image is included, and the percentage error of each solution relative to the numerical solution is presented in Table 1, Table 2, Table 3, Table 4.

**Table 1:** *Errors in HFAM solution compared to numerical solution for Case-I when*A=0.3,g=9.81,L=2.5,m=45,κ=4.5,J=260..

Time	HFAM Results	Numerical Results	Error in Percentage
0	0.3	0.3	0
5	0.202265	0.202477	0.104927
10	−0.0272599	−0.0272996	0.145518
15	−0.239023	−0.239177	0.0646165
20	−0.295046	−0.295069	0.00764343
25	−0.158826	−0.159067	0.150996
30	0.0808794	0.0809882	0.134342
35	0.267887	0.267972	0.0316792
40	0.280348	0.280431	0.0298456
45	0.110143	0.110371	0.20663
50	−0.131828	−0.131978	0.113547

**Table 2:** *Errors in HFAM solution compared to numerical solution for Case-II when*A=0.25,g=9.81,L=2.1,m=35,κ=8,δ=18,J=300..

Time	HFAM Results	Numerical Results	Error in Percentage
0	0.25	0.25	0.
5	0.164911	0.164957	0.02783
10	0.108767	0.108879	0.103286
15	0.0717265	0.0718151	0.123338
20	0.0472931	0.047315	0.0462712
25	0.0311783	0.0311335	0.143954
30	0.0205514	0.0204585	0.454051
35	0.0135446	0.0134254	0.887812
40	0.00892541	0.00879796	1.44871
45	0.00588064	0.00575739	2.1408
50	0.00387397	0.00376227	2.96879

**Table 3:** Errors in HFAM solution compared to numerical solution for Case-III when A=0.2,g=9.81,L=2.1,m=29,κ=12,F=20,λ=60°,J=230..

Time	HFAM Results	Numerical Results	Error in Percentage
0	0.2	0.2	0.
5	0.101762	0.101906	0.1415
10	−0.0948027	−0.0946398	0.172137
15	−0.193393	−0.19299	0.208759
20	−0.0958415	−0.0956213	0.230359
25	0.099737	0.100083	0.346179
30	0.197091	0.197297	0.10459
35	0.0981166	0.0985024	0.391668
40	−0.0990003	−0.0985635	0.44311
45	−0.19793	−0.197356	0.290694
50	−0.100489	−0.100136	0.352055

**Table 4:** Errors in HFAM solution compared to numerical solution for Case-IV when A=0.25,g=9.81,L=2.6,m=24,κ=8,δ=12,F=25,λ=35°,J=220..

Time	HFAM Results	Numerical Results	Error in Percentage
0	0.25	0.25	0.
5	−0.0598265	−0.0584698	2.3202
10	0.182523	0.181906	0.339283
15	−0.0961173	−0.0950521	1.12061
20	0.146364	0.145537	0.5683
25	−0.10484	−0.104054	0.755293
30	0.1222	0.121397	0.661036
35	−0.0994338	−0.0988527	0.587915
40	0.101589	0.10093	0.65324
45	−0.0861919	−0.0857746	0.486496
50	0.0810748	0.0805991	0.590268

The subfigures labeled as (a) in Fig. 2 display the HFAM solution (11) alongside the corresponding numerical solutions of the nonlinear jet engine vibration system for different sets of parameter values and initial conditions, particularly in cases where no damping and driving forces are present. Subfigures (b) provide magnified views of specific sections within the corresponding subfigures (a). Upon examining the zoomed-in sections of the figures, it becomes evident that there is negligible difference between the derived solution and the numerical results. Additionally, Table 1 presents the fact that the errors in the derived solution are exceedingly small, very close to 0 %, when compared to the numerical results.

For various sets of parameters values and initial conditions Fig. 3 portrays the (a) subfigures, which present the HFAM outcome (13) along with their corresponding numerical counterparts for the nonlinear jet engine vibration system in the case of damping and devoid of driving forces. Simultaneously, the (b) subfigures provided a detailed perspective by zooming in on specific regions of the (a) subfigures, which demonstrated the precision of the derived solution, while also showcasing its reliability and effectiveness in accurately representing the underlying dynamics. Furthermore, Table 2 reveals that the most notable two errors linked to the acquired solution is around 3 % at time 50 in comparison to the numerical results, while all remaining errors remain below 2.15 %.

Subfigures (a) in Fig. 4 depict a comparison between the numerical results and obtained solution (15) using HFAM for three distinct parameter sets and initial conditions. This comparison is made under the influence of a driving force without the presence of damping. Conversely, subfigures (b) within subfigures (a) offer magnified views of specific segments, highlighting the accuracy of the derived solutions. This magnified perspective also emphasizes the method's robustness, efficiency, and efficacy. Simultaneously, the data presented in Table 3 illustrates that the errors associated with the derived solution consistently remain at notably low levels for all time values, which are not more than 0.45 %.

In Fig. 5, the subfigures marked as (a) display the HFAM solution (17) along with their corresponding numerical counterparts for the nonlinear jet engine vibration system. Subfigures (b) offer close-up views of specific sections of the related subfigures (a), showcasing various parameter values and initial conditions, especially when damping and driving forces are present. By closely inspecting the magnified parts of the figures, it becomes evident that exceedingly minor distinctions are observable between the two solutions. Additionally, Table 4 discloses that the most significant error associated with the obtained solution is around 2.32 % when time is 5 with respect to numerical results, and all other errors are less than 1.15 %.

## 5 Conclusion

This study demonstrates the effectiveness of HFAM in addressing the complex jet engine vibration equations across various operational conditions. Careful comparisons with numerical solutions for nonlinear phenomena, including damping and driving forces, validate the accuracy and computational efficiency of HFAM. These achievements hold significant implications for aerospace engineering, providing insights into vibration mitigation and performance optimization. Beyond the aerospace domain, our research underscores the potential of HFAM as a versatile analytical tool for intricate engineering challenges. As our findings transcend disciplines, they encourage the exploration of the adaptability of HFAM. Implementing our results in the industry could enhance jet engine design and operation. Future research might delve into nonlinearities and transient behaviors. Collaborative efforts between academia and industry could provide real-world validation. The proficiency of HFAM in solving nonlinear oscillation problems, including damping and driving forces, solidifies its reputation for delivering rapid, precise solutions, essential for engineering analysis.

## Ethical approval

Not applicable.

## Consent for publication

Not applicable.

## Availability of data and materials

No data used for research described in the article.

## Funding

The author(s) received no financial support for the research, authorship, and/or publication of this article.

## Additional information

No additional information is available for this paper.

## CRediT authorship contribution statement

**M. Abul Kawser:** Conceptualization, Formal analysis, Methodology, Supervision, Writing – original draft, Writing – review & editing. **Md. Abdul Alim:** Conceptualization, Formal analysis, Methodology, Writing – original draft, Writing – review & editing. **Nazmul Sharif:** Conceptualization, Methodology.

## Declaration of competing interest

The authors declare that they have no known competing financial interests or personal relationships that could have appeared to influence the work reported in this paper.
